# Government subsidies for GLP-1 agonists in mental health patients with metabolic syndrome: A call for quality-of-life improvement and cost reduction

**DOI:** 10.1177/10398562251313696

**Published:** 2025-01-13

**Authors:** Wole Akosile

**Affiliations:** Brisbane, QLD, Australia

Dear Editor,

I am writing to express the importance of government subsidies for GLP-1 agonists in people living with mental illnesses with metabolic syndrome or obesity. This proposal offers significant benefits, including improved quality of life and increased life expectancy. These medications can help prevent costly cardiometabolic events and reduce hospital stays, leading to substantial economic savings. By addressing both metabolic and mental health issues, GLP-1 agonists provide a comprehensive approach to treatment that can alleviate the overall burden on the healthcare system.^1^ GLP-1 agonist medications, such as semaglutide, exenatide, and liraglutide, are primarily indicated for the management of Type 2 diabetes mellitus, particularly in patients with specific circumstances such as obesity or those who have not achieved adequate control with other treatments.

People living with mental illnesses often face a heightened risk of metabolic syndrome due to a combination of psychotropic medications, sedentary lifestyles, and poor dietary habits.^2,3^ The link between metabolic syndrome and an increased risk of cardiovascular disease is well-established.^2,3^ Given the prevalence of mental health conditions and their impact on overall health, addressing metabolic syndrome in this population is crucial.^2,3^

GLP-1 agonists have shown promise in improving metabolic parameters and reducing cardiovascular risk in those with and without diabetes. GLP-1 agonists not only enhance glucose control but also have favourable effects on weight, blood pressure, and lipid profiles.^4^ In people living with mental illnesses, the potential benefits extend beyond metabolic parameters. Improved metabolic health has been associated with better mental health outcomes, leading to enhanced overall well-being and quality of life.^5^

Furthermore, the economic implications of subsidizing GLP-1 agonists for this population are substantial.^5^ By preventing future cardiac events and reducing the need for hospitalization, there is a potential for significant cost savings within the healthcare system.

In light of these considerations, I urge the medical community and policymakers to recognize the potential impact of subsidizing GLP-1 agonists for people living with mental illnesses with metabolic syndrome.

Ethical approval was not required for this study.
